# Isolation of Aflatoxin B1 from Moldy Foods by Solid-Phase Extraction Combined with Bifunctional Ionic Liquid-Based Silicas

**DOI:** 10.1155/2018/8427580

**Published:** 2018-11-08

**Authors:** Luwei Fang, Minglei Tian, Xuemin Yan, Wei Xiao

**Affiliations:** College of Chemistry and Environmental Engineering, Yangtze University, Jingzhou, Hubei 434023, China

## Abstract

A solid-phase extraction method was developed by using new bifunctional ionic liquid-based silicas as sorbents to isolate aflatoxin B1 from moldy corn and peanut. Firstly, according to the adsorption efficiency, two sorbents imidazolium chloride-butylimidazolium chloride-based silica (Sil@BIm-Im) and imidazolium chloride-hexylimidazolium chloride-based silica (Sil@HIm-Im) were selected. The RSM was introduced to optimize adsorption conditions such as methanol/water ratio, time, and pH. Sil@HIm-Im, which had the highest adsorption efficiency, was used in SPE as a sorbent. After 2.0 mL of loading samples, washing solvents were optimized as 6.0 mL and 4.0 mL of water for corn and peanut, 2.0 mL of acetonitrile, and 3.0 mL of methanol. 3.0 mL of methanol/acetic acid (2.0% vol.) was investigated as an elution solvent. Finally, 0.009 *μ*g/g and 0.023 *μ*g/g of aflatoxin B1 were obtained in corn and peanut extract with recoveries of 80.0%–103.3% and RSDs of 2.37%–6.58%.

## 1. Introduction

Aflatoxins (AFs) such as B1, B2, G1, and G2 are natural secondary metabolites that are produced by molds [1]. AFs are found as contaminants in various agricultural commodities such as cereals, tree nuts, groundnut, and cottonseed [2]. They are considered the most serious threat to public health due to their carcinogenic and hepatotoxic, teratogenic and mutagenic effect in human and animals [3–5].

Various methods have been published to reduce or detoxify aflatoxins; however, no treatment has been found to be effective against aflatoxins [6]. Several chemicals such as oxidizing and reducing agents, acids or bases, salts, and chlorinating agents have been investigated to degrade or detoxify aflatoxin; however, the results of these treatments were not satisfactory [7–9]. Hence, some researchers applied various methods to detect AFs. Furthermore, chromatographic methods were applied to adsorb AFs. L. Sun reported a fluorescence anisotropy (FA) assay for detection of AFB1 binding [10]. Another pELISA method exhibited a high sensitivity for AFB1 visual detection with a limit of 12.5 pg/mL [11]. Mazaheri used HPLC with a C_18_ column to detect aflatoxins in 71 rice samples, and 1.89 ng/g of AFB1 was found in all samples [12]. Herzallah detected aflatoxins in several food products using HPLC, and the contents were in the range from 0.15 to 8.32 *μ*g/L [13]. Zahoor and Khan prepared magnetic carbon nanocomposites to adsorb aflatoxin from poultry feed. The adsorption time was 115.0 min at pH = 3.0 while 150.0 min at pH = 7.0 [14].

In the previous method, large amount of solvents especially organic solvents were regulated as another kind of pollutants in the process. So, solid-phase extraction (SPE) as one of the most convenient and high-performance separation technologies can help minimize the use of organic solvents [15]. In order to adsorb AFs, the selection of the sorbent is a key point because it can control analytical parameters such as adsorbed amount, selectivity, and affinity.

Ventura et al. used an Oasis HLB SPE cartridge with HPLC/MS to detect four aflatoxins in a herb, 2.0 mL of methanol/water (30 : 70, vol%) was used as a washing solvent, and 4.0 mL of methanol/water (80 : 20, vol%) was used as an elution solvent [16]. Liu et al. synthesized an aptamer-functionalized magnetic agarose microsphere and applied it in SPE to adsorb AFB1 and AFB2 with an HPLC-fluorescence detection. The binding capacity is 350.0 ng/g for AFB1 and 384.0 ng/g for AFB2, and 1.0 mL of methanol/eluting buffer (20 : 80, v/v) was used as an elution solvent [17].

However, low- or nontoxic solvent/sorbents are still needed urgently. Therefore, ionic liquids (ILs) were introduced and received many attentions. ILs, as green solvents with excellent chemical properties [18], are already employed as a modifier in the fields of analytical chemistry [19, 20], sample preparation [21, 22], organic synthesis [23–25], liquid-phase extraction [26–28], and chromatographic separations [29]. The characteristics, such as hydrophobicity, miscibility with several inorganic/organic solvents, and *π* − *π* interactions between analyte and functional groups of the ionic liquids, are widely applied [30]. Some reports have examined the application of obtained ionic liquid-modified materials to separate familiar organic compounds [31, 32].

In this research, AFB1 was adsorbed using bifunctional ionic liquid-based silicas. During the adsorption, surface response methodology (RSM) with the Design-Expert software optimized three variable conditions such as methanol/water ratio, adsorption time, and pH. Finally, the sorbent isolated AFB1 from extracts of moldy corn and peanut with the SPE method. The work is expected to develop the SPE method based on unique sorbent and extend their potential application in the separation and purification of other compounds.

## 2. Materials and Methods

### 2.1. Materials

Silica (15–31 *μ*m), (3-chloropropyl)trimethoxysilan (98%), imidazole (99%), 1-methylimidazole (99%), 1-ethylimidazole (98%), 1-butylimidazole (98%), 1,2-dichloroethane (99%), 1,4-dichlorobutane (98%), 1,6-dichlorohexane (99%), and aflatoxin B1 (>98%) were purchased from Aladdin Inc. (Shanghai, China). The HPLC grade of acetonitrile and methanol was from CINC High Purity Solvents Co. Ltd. (Shanghai, China). Other organic solvents such as acetone, ethanol, toluene, and triethylamine were supplied by Beilian Company (Tianjing, China), and purities were higher than 99.0%. Ultrapure water was produced by a purification machine (UPH-I-5, Youpu, China), and all organic solvents should be filtered before use.

### 2.2. Preparation of Bifunctional IL-Based Silicas

**Figure 1:**
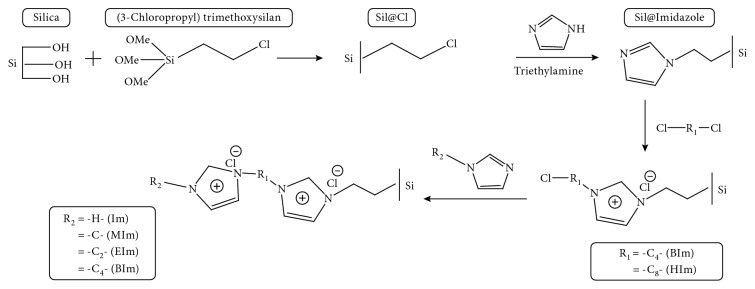
Synthesis process of IL-based silicas.

Silica was activated by stirring in 10.0% vol. HCl aqueous solution for 24 hr and washed by water until pH = 7.0. Then, the activated silica was synthesized by the following steps shown in Figure 1. Firstly, chlorine was modified on the silica surface by adding (3-chloropropyl)trimethoxysilane as a coupling compound. Subsequently, 30.0 g of activated silica, 40.0 mL of (3-chloropropyl)trimethoxysilane, and 80.0 mL of toluene were well stirred and heated to 100°C in a flask for 12 hr. The obtained powder (3-chloropropyl-based silica, Sil@Cl) was then washed with ethanol and fully dried. All obtained Sil@Cl, 25.0 g of imidazole, 20.0 mL of triethylamine, and 80.0 mL of toluene were mixed and synthesized in a flask for 8 hr under 100°C, and then, the silica@imizadole was obtained. After that, three flasks were prepared, and 8.0 g of silica@imizadole was mixed with 80.0 mL of toluene in each flask. Then, 10.0 mL of 1,4-dichlorobutane and 1,6-dichlorohexane were added into each flask, respectively. After all flasks were heated to 100°C for 12 hr, chlorinated IL-based silicas Sil@BIm-Cl and Sil@HIm-Cl were obtained. At last, 1.5 g of these two obtained silicas and different types of imidazoles were synthesized in 50.0 mL of toluene: Sil@BIm-Im (imidazolium chloride-butylimidazolium chloride-based silica), Sil@BIm-MIm (methyimidazolium chloride-butylimidazolium chloride-based silica), Sil@BIm-EIm (ethylimidazolium chloride-butylimidazolium chloride-based silica), Sil@BIm-BIm (double butylimidazolium chlorides-based silica), Sil@HIm-Im (imidazolium chloride-hexylimidazolium chloride-based silica), Sil@HIm-MIm (methylimidazolium chloride-hexylimidazolium chloride-based silica), Sil@HIm-EIm (ethylimidazolium chloride-hexylimidazolium chloride-based silica), and Sil@HIm-BIm (butylimidazolium chloride-hexylimidazolium chloride-based silica).

### 2.3. Apparatus

The characteristics of all obtained IL-based silicas were analyzed by FT-IR (Nicolet 6700, Thermo Fisher, Waltham, USA) in the range of 400–4000 cm^−1^ with a scan rate of 20 scans/min and TGA (Labsys evo, Setaram, Caluire, France) with a heating rate of 10°C/min under N_2_. The chromatographic conditions were as follows: HPLC (LC3000, CXTH, Beijing, China) with a TC-C18 column (4.6 × 250 mm, 5 *μ*m, Agilent, USA), and acetonitrile/water (40 : 60, v/v) was used as the mobile phase. The flow rate, UV wavelength, injection volume, and column oven temperature were 0.8 mL/min, 365 nm, 5.0 *μ*L, and 35°C, respectively.

### 2.4. Adsorbent Selection and Optimization of Conditions by RSM

First of all, 0.1 g of all IL-based silicas mixed with 1.0 mL of 0.1 *μ*g/mL of aflatoxin B1 in methanol within 5.0 min at ambient temperature by the liquid-solid adsorption method and the adsorption amounts of all sorbents were detected by analyzing the concentration in liquid layers.

Then, the adsorption conditions were optimized by Design-Expert software (v.8.0.6, Stat-Ease, Inc, Minneapolis, USA). The BBD model in RSM was used, and according to physical and chemical properties of aflatoxin B1, three major factors such as methanol/water ratio, adsorption time, and pH, which can significantly affect the maximum adsorption amount, were selected. Three factors of the BBD were designated A, B, C and divided into three levels in Table 1. The codes −1, 0, and +1 were assigned as low, intermediate, and high values, respectively.

### 2.5. Procedure of SPE

**Figure 2:**
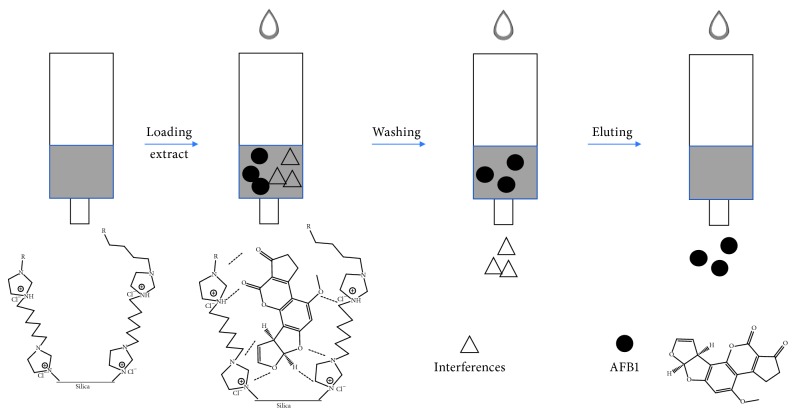
Process of SPE and adsorption mechanism.

Figure 2 shows the process of SPE. 0.1 g of IL-based silicas was packed into an empty SPE cartridge (Ø 0.9 cm, Alltech, Deerfield, IL, USA). The SPE-Sil@IL cartridge was conditioned with 10.0 mL methanol. After the sample solution loading on the sorbents, hexane, water, acetonitrile, and methanol were used as washing solvents and methanol/acetic acid (2.0% vol.) as the elution. The solvents collected from the washing and elution steps were analyzed by HPLC.

### 2.6. Preparation of Moldy Food Samples

Peanut and corn were stored under a high moisture aerobic ambient at 30°C for a month. Then, the moldy samples were ground to powder, and 25.0 g of them were dipped in 50.0 mL of methanol over 24 hr. The obtained extracts were filtered and stored at 4°C for further analysis.

## 3. Results and Discussion

### 3.1. Characterization

**Figure 3:**
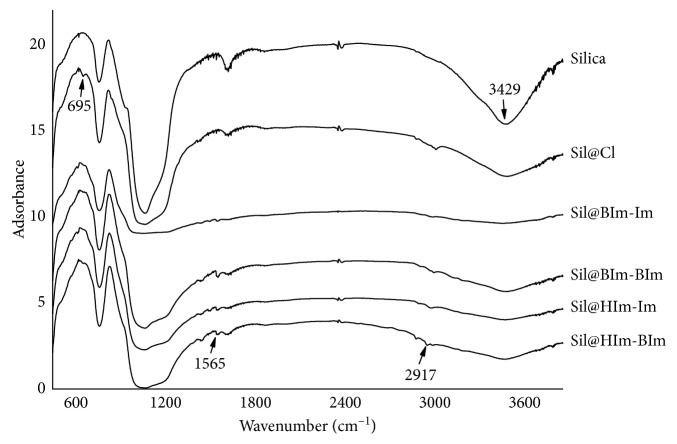
FT-IR of silica and IL-based silicas.

In the FT-IR spectra (Figure 3), silica showed a wide peak at 3429 cm^−1^ and this peak belonged to the –OH group. In Sil@Cl, the –OH peak decreased and C-Cl peak at 695 cm^−1^ appeared. After modification, the IL-based silicas showed the appearance of peaks nearby 1565 cm^−1^ which is the fingerprint region of IL groups, and the C-Cl peak disappeared. Moreover, the vibration range of C-H group in alkanes is 2850–3000 cm^−1^. So, the peaks at 2917 cm^−1^ belonged to the carbon chain on the IL group, also the peak area increased with the carbon chain length increasing [33, 34].

**Figure 4:**
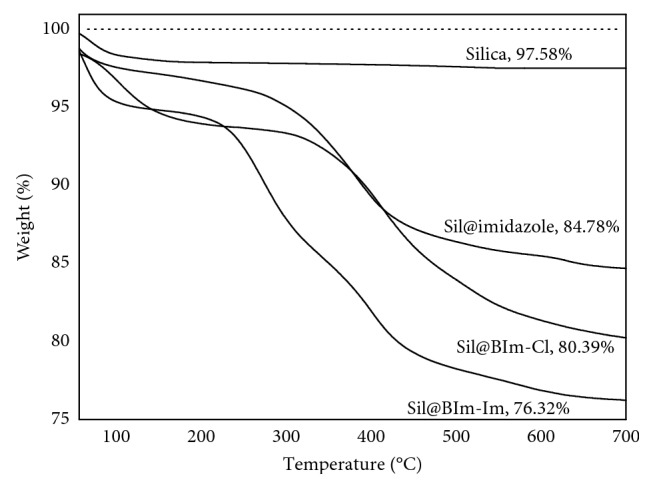
TGA for silica and IL-based silicas.

Thermogravimetric analysis (TGA) can determine the thermostability of the additional groups on silica with the weight loss.  Figure 4 shows that, under 700°C, the sorbents contained more functional IL groups that lost more weight from 12.80 to 21.26%. Both FT-IR and TGA results indicated that the bifunctional IL groups were successfully modified on silica.

### 3.2. Adsorbent Selection and Optimization of Conditions

First of all, the calibration curves were constructed using the chromatographic peak areas measured at several increasing concentrations of aflatoxin B1 ranging from 0.001 to 10.0 *μ*g/mL. Good linearity was obtained, and the linear correlation equations were *y* = 1 × 10^−7^*x −* 4643 (*R*^*2*^ = 0.994) (*y* is peak area and *x* is the concentration>).

Table 2 shows the adsorption amount of aflatoxin B1 on different sorbents. Because of the structure of aflatoxin B1, it was a hydrophobic compound and contained several oxygen groups, hydrogen groups, and benzene rings. So, between the IL group and aflatoxin B1, there were hydrogen and *π* − *π* interactions. Also, the hydrophobicity of the carbon chains between two IL groups had strong interaction on aflatoxin B1. In this case, IL-based silicas obtained much more adsorbed amount than C_18_. In addition, the carbon chain on the end of the IL group also had hydrophobicity. However, when the chain was too long, it would swing and may cover the inner IL group to weaken the effect and break chemical bonds. So, from the results, the adsorption amounts on IL with long carbon chain decreased. Hence, Sil@BIm-Im and Sil@HIm-Im were selected, and the optimized adsorption conditions of them were design by RSM.

According to the results from the designed experiments by software, the model proposed for the response of the adsorbed amounts of aflatoxin B1 was determined as Equations (1) and (2):

(1) Y=0.32+0.082 ∗ A+0.083 ∗ B+0.082 ∗ C+0.02 ∗ AB+0.026 ∗ AC+0.034 ∗ BC+0.041 ∗ A2+0.015 ∗ B2+0.0065 ∗ C2of  Sil@BIm−Im,

(2) Y=0.23+0.11 ∗ A+0.043 ∗ B+0.15 ∗ C+0.019 ∗ AB+0.03 ∗ AC+0.035 ∗ BC+0.04178 ∗ A2+0.1 ∗ B2+0.015 ∗ C2of  Sil@HIm−Im.

For both equations, the model *F* values higher than 39.59 and Prob>*F* less than 0.05 indicated that the models were significant. The value of *R*^2^ = 0.9941 for Equation (1) and *R*^2^ = 0.9807 for Equation (2) showed a high correlation degree between the observed and predicted adsorbed amounts. The software gave the following optimized conditions: methanol/water (96 : 4, vol%), 26.63 min of adsorption, and pH = 6.5 of Sil@BIm-Im; methanol/water (99 : 1, vol%), 29.73 min of adsorption, and pH = 6.8 of Sil@HIm-Im. Under the optimized conditions, the adsorption amount of Sil@HIm-Im is 13.46% higher than Sil@BIm-Im.

**Figure 5:**
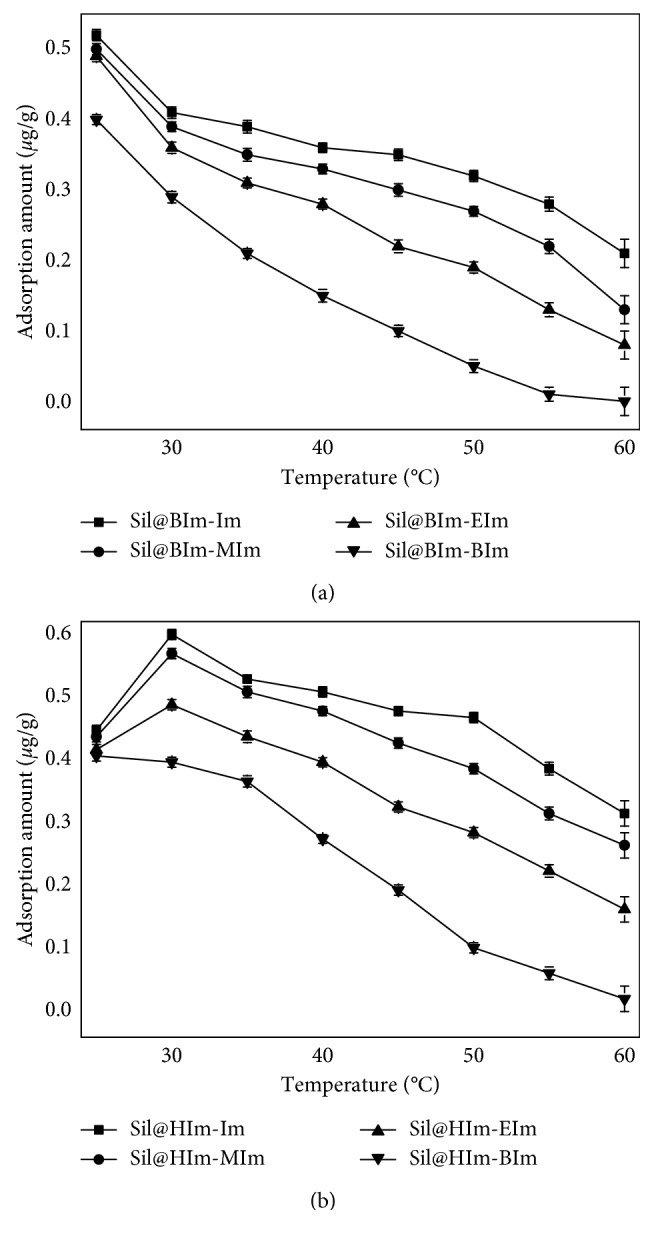
Adsorption amounts of IL-based silicas at different temperatures.

Then, the ambient temperature was changed on all sorbents. In Figure 5, the results showed that under high temperature, the adsorption efficiencies of all sorbents decreased. Hence, the appropriate temperature was 25°C for Sil@BIm-Im and 30°C for Sil@HIm-Im, respectively. Summarizing all above results, Sil@HIm-Im showed higher efficiency than other sorbents, and it was selected for the next step.

### 3.3. Isolation of Aflatoxin B1 from Extracts by SPE

Two microliters of the extract solution was loaded onto Sil@HIm-Im with 30.0 min adsorption. After loading, a washing step should be applied immediately to reduce the matrix interferences in separation.

First, 1.0 mL of hexane, water, ethanol, acetonitrile, and methanol were used to remove hydrophobic/hydrophilic and organic interference without aflatoxin B1. However, hexane and ethanol could remove almost nothing from the sorbent. So water and acetonitrile were selected. Then, the volumes of water and acetonitrile were increased to remove more interferences. The corn extract contained more water-soluble substances, so, it needed more water than the peanut extract. According to the obviously decreasing of interferences peak area in chromatograms, 6.0 mL and 4.0 mL of washing water was used for corn and peanut extracts, respectively. And 2.0 mL of acetonitrile was used for both extracts. Large amount of interferences was washed out by methanol; however, when the volume was larger than 3.0 mL, a small amount of aflatoxin B1 could be removed. So, 3.0 mL of methanol was selected.

**Figure 6:**
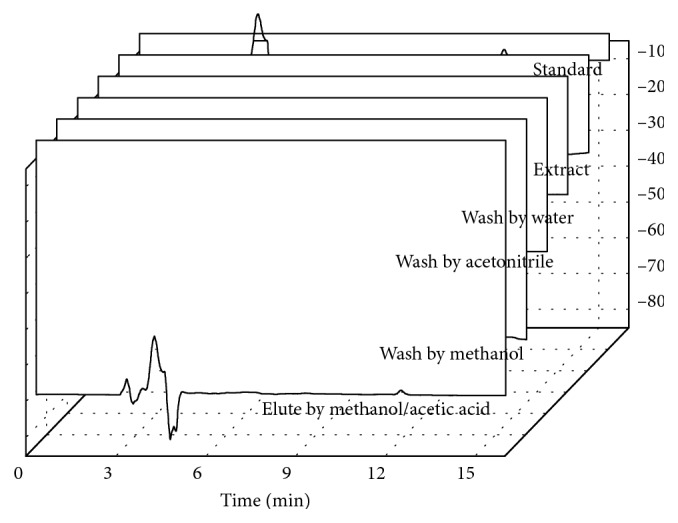
Chromatograms of standard, extract, washing, and elution solutions.

Then, methanol/acetic acid (2.0% vol.) and acetonitrile/acetic acid (2.0% vol.) with a higher eluting strength were used as elution solvents. The results showed that 3.0 mL of methanol/acetic acid (2.0% vol.) had more efficiency than acetonitrile/acetic acid (2.0% vol.) and could elute all aflatoxin B1 from the sorbent.  Figure 6 reveals that the aflatoxin B1 was successfully isolated from the extract. Finally, 0.009 *μ*g/g and 0.023 *μ*g/g of aflatoxin B1 were obtained in corn and peanut extracts, respectively.

### 3.4. Repeatability and Stability on the Performance

In corn and peanut extract solutions, a certain concentration of aflatoxin B1 was spiked and analyzed to calculate the recoveries. Assays of repeatability calculated as relative standard deviations (RSDs) were performed with Sil@HIm-Im by SPE process 5 times in a 5-day period. The recoveries and RSDs are presented in Table 3. Good recoveries of 80.0%–103.3% and RSDs of 2.37%–6.58% were obtained. The results mean that the sorbent is reliable for real samples.

## 4. Conclusions

In this study, bifunctional ionic liquid-based silicas were created and applied in SPE to isolate aflatoxin B1 from moldy corn and peanut extracts. According to the adsorption amount by the standard solution, more aflatoxin B1 was adsorbed on Sil@BIm-Im and Sil@HIm-Im. With the designed experiment of RSM, Sil@HIm-Im was selected as the SPE sorbent, and the optimized conditions were methanol/water (99 : 1, vol%), 29.73 min of adsorption, and pH = 6.8. In addition, the ambient temperature was evaluated, and the results proved Sil@HIm-Im to be the SPE sorbent. Finally, washing by water and acetonitrile and eluting by 3.0 mL of methanol/acetic acid (2.0% vol.), 0.009 *μ*g/g and 0.023 *μ*g/g of aflatoxin B1 were obtained in corn and peanut extracts. The low deviation error, stable characteristics, and application demonstrated the sorbent to be a viable alternative tool for further researches.

## Figures and Tables

**Table 1:** Levels of independent variables used in BBD.

Variables	Level		
	−1	0	+1
Methanol/water (v : v) (A)	20/80	60/40	100/0
Adsorption time (min) (B)	5.0	17.5	30.0
pH (C)	2.0	4.5	7.0

**Table 2:** Adsorption amounts of aflatoxin B1 on 0.1 g of different sorbents within 5 min at ambient temperature.

Sorbent	Loading volume	Loading concentration	Adsorption amount (*μ*g/g)
Sil@BIm-Im	1.0 mL	0.1 *μ*g/mL in methanol	0.33
Sil@BIm-MIm			0.32
Sil@BIm-EIm			0.27
Sil@BIm-BIm			0.19
Sil@HIm-Im			0.32
Sil@HIm-MIm			0.31
Sil@HIm-EIm			0.25
Sil@HIm-BIm			0.18
C_18_			0.19

**Table 3:** The recoveries and RSDs in real samples (*n*=5).

Sample	Added (*μ*g/g)	Founded (*μ*g/g)	Recovery (%)	Intraday RSD (%)	Interday RSD (%)
Corn	0.000	0.009	—	2.37	3.13
	0.010	0.009	90.0	4.35	4.56
	0.030	0.037	93.3	4.58	4.63
	0.050	0.058	98.0	5.44	5.86
	0.100	0.111	102.0	5.87	6.03

Peanut	0.000	0.023	—	2.77	3.29
	0.010	0.031	80.0	3.86	3.89
	0.030	0.054	103.3	4.24	4.97
	0.050	0.073	100.0	4.21	4.68
	0.100	0.121	98.0	6.01	6.58

## Data Availability

The data given in Introduction to support the findings of this study have been deposited in the repository. All data given in Materials and Methods and Results and Discussion to support the findings of this study are included within the article.
